# Differential Inflammatory and Immune Response to Viral Infection in the Upper-Airway and Peripheral Blood of Mild COVID-19 Cases

**DOI:** 10.3390/jpm14111099

**Published:** 2024-11-09

**Authors:** Malena Gajate-Arenas, Omar García-Pérez, Angélica Domínguez-De-Barros, Candela Sirvent-Blanco, Roberto Dorta-Guerra, Alma García-Ramos, José E. Piñero, Jacob Lorenzo-Morales, Elizabeth Córdoba-Lanús

**Affiliations:** 1 Instituto Universitario de Enfermedades Tropicales y Salud Pública de Canarias (IUETSPC), Universidad de La Laguna, 38029 La Laguna, Tenerife, Spain; alu0101017877@ull.edu.es (M.G.-A.); ogarciap@ull.edu.es (O.G.-P.); alu0100898019@ull.edu.es (A.D.-D.-B.); alu0101234656@ull.edu.es (C.S.-B.); rodorta@ull.edu.es (R.D.-G.); alu0101204734@ull.edu.es (A.G.-R.); jpinero@ull.edu.es (J.E.P.); 2 Centro de Investigación Biomédica en Red de Enfermedades Infecciosas (CIBERINFEC), Instituto de Salud Carlos III, 28029 Madrid, Spain; 3 Departamento de Matemáticas, Estadística e Investigación Operativa, Facultad de Ciencias, Sección de Matemáticas, Universidad de La Laguna, 38200 La Laguna, Tenerife, Spain; 4 Departamento de Obstetricia y Ginecología, Pediatría, Medicina Preventiva y Salud Pública, Toxicología, Medicina Legal y Forense y Parasitología, Facultad de Ciencias de la Salud, Universidad de La Laguna, 38200 La Laguna, Tenerife, Spain

**Keywords:** COVID-19, SARS-CoV-2, upper-airway, blood, saliva, immune response, gene expression

## Abstract

Background/Objectives: COVID-19 is characterised by a wide variety of clinical manifestations, and clinical tests and genetic analysis might help to predict patient outcomes. Methods: In the current study, the expression of genes related to immune response (*CCL5*, *IFI6*, *OAS1*, *IRF9*, *IL1B*, and *TGFB1*) was analysed in the upper airway and paired-blood samples from 25 subjects infected with SARS-CoV-2. Relative gene expression was determined by RT-qPCR. Results: *CCL5* expression was higher in the blood than in the upper airway (*p* < 0.001). In addition, a negative correlation was found between *IFI6* and viral load (*p* = 0.033) in the upper airway, suggesting that the *IFI6* expression inhibits the viral infection. Concerning sex, women expressed *IL1B* and *IRF9* in a higher proportion than men at a systemic level (*p* = 0.008 and *p* = 0.049, respectively). However, an increased expression of *IRF9* was found in men compared to women in the upper airway (*p* = 0.046), which could be due to the protective effect of *IRF9*, especially in men. Conclusions: The higher expression of *CCL5* in blood might be due to the key role of this gene in the migration and recruitment of immune cells from the systemic circulation to the lungs. Our findings confirm the existence of sex differences in the immune response to early stages of the infection. Further studies in a larger cohort are necessary to corroborate the current findings.

## 1. Introduction

Coronavirus Disease 2019 (COVID-19), a respiratory illness, is caused by Severe Acute Respiratory Syndrome Coronavirus 2 (SARS-CoV-2). Coronaviruses constitute a family of single-stranded RNA viruses, well-known for causing respiratory infections. Coronaviruses can be classified in 4 genera, *Alphacoronavirus*, *Betacoronavirus*, *Deltacoronavirus*, and *Gammacoronavirus*. *Alphacoronavirus* and *Betacoronavirus* characteristically infect mammals, while *Deltacoronavirus* and *Gammacoronavirus* mostly infect birds. The most pathogenic coronaviruses to humans are within the *Betacoronavirus* genus, including SARS-CoV, MERS-CoV, and SARS-CoV-2 [1].

Of the three viruses mentioned before, SARS-CoV was the first to cause an epidemic outbreak in China (2002); the epidemic was contained, and cases have not been reported since 2004. MERS-CoV was first detected in Jordan in 2012, and currently causes persistent endemics in Middle Eastern countries, eventually spreading to countries outside this region. Finally, SARS-CoV-2 emerged in China in 2019, and spread globally, causing a pandemic outbreak [2].

SARS-CoV-2 infection is transmitted by aerosols and close contact. The infections start in the respiratory tract, where the viral replication through the epithelial cells promotes the migration of the virus to the lungs. During this process, an immune response is promoted, but an excessive and non-coordinated response triggers damage in the lungs instead of relieving the infection [3,4,5,6,7]. This impaired immune response is characterised by the increase in the proinflammatory cytokines which promotes the influx of immune cells from circulation to the lungs. As a result, patients can suffer respiratory distress syndrome, respiratory failure, multi-organ failure, and death [8].

As a result of the complexity of the interaction between the virus and the host, COVID-19 presents a wide range of clinical manifestations, from asymptomatic or mild cases to severe forms of the disease that require intensive patient care [3,9]. The predisposition to suffering severe COVID-19 depends on several factors like age, sex, or underlying diseases. Most severe cases occur in patients over their sixties and mostly in men [3,5,10,11]. Moreover, pathologies like diabetes, obesity, or cardiorespiratory illness increase the severity risk [12,13]. Clinical features are not enough to explain the complex pathogenesis of SARS-CoV-2 and its clinical manifestations, which clearly suggests that individual genetic variability plays a main role in the course of the infection.

There are different ways to study genetic variability. One of the most common is single nucleotide polymorphisms (SNPs) analysis, which allows disease-gene association studies [14]. The essence of these investigations is looking for mutations in certain genes of interest. Much research has focused on the viral cycle, looking for mutations in genes like *ACE2* or *TRMPRSS2* that might explain part of the susceptibility to SARS-CoV-2 infection [15]. Genes related to the immune system are also interesting to analyse, as mutations in genes related to antigen recognition or the inflammatory process could shed light on failures during the immune response [16]. However, it is necessary to clarify how the genotype leads to the phenotype and explain the genetic variability beyond DNA. Therefore, many researchers have also focused on the analysis of mRNA expression that may reveal alterations in important genes [17,18]. Alterations in RNA expression have been related to the development of either chronic or infectious diseases [19,20]. Therefore, the analysis of the expression of genes involved in viral pathogenesis may shed light on the process and may help define the patient’s prognosis.

Genes related to immune response or inflammatory pathways have been found altered in their expression by SARS-CoV-2 infection and have been associated with worse outcomes [21,22,23]. In the current study, we analyse different genes involved in the immune response. Among them, transforming growth factor beta 1 (*TGFB1)* and interleukin 1 beta (*IL1B)* are cytokines that promote an inflammatory response [24,25], while the C-C motif chemokine ligand (*CCL5*) is a chemoattractant that induces the migration and recruitment of several immune cells [26]. The interferon regulatory factor 9 (*IRF9)* promotes negative feedback in the interferon response, and its deficiency causes excessive inflammation in viral infections [27]. The interferon alpha inducible protein 6 (*IFI6)* is a mitochondria-target protein involved in the regulation of the apoptotic process [28]. The 2′-5′-oligoadenylate synthetase 1 (*OAS1*) encodes for an antiviral enzyme which interferes in viral replication [29].

Furthermore, it is important to consider that gene expression is not homogenous in the human body, and samples from different types of tissues might show different expression levels. The upper airway is the first target of SARS-CoV-2 and could give us information about the local immune response developed for controlling the initial infection [30,31]. On the other hand, the analysis of peripheral fluid, like serum or whole blood, might give insights of the infection progression at a systemic level [32].

The main objective of the current research was to analyse and compare the differential expression profile of inflammatory and immune-related genes from the upper airways and peripheral blood in mild cases infected with SARS-CoV-2. Based on the existing literature and according to a previous study conducted by our team, the genes *CCL5*, *IFI6*, *OAS1*, *IRF9*, *IL1B*, and *TGFB1* were analysed in the current research. In the mentioned study, we found a differential expression of genes related to antiviral activity and immune response in the early stages of the infection in the upper airways of mild COVID-19 cases [33]. Therefore, there is a need to clarify if this local immune response is extrapolated to a systemic level. Studying genes related to immune response can provide essential information about the virus pathogenesis and identify potential biomarkers that can enhance the patient’s diagnosis, prognosis, and treatment.

## 2. Materials and Methods

### 2.1. Patients and Study Samples

The study included 25 male and female cases restricted to mild forms of COVID-19 which did not require more than ambulatory care. Individuals had a positive diagnosis of SARS-CoV-2 infection by qPCR and were recruited between 2022 and 2023 at IUETSPC-University of La Laguna in Tenerife (Canary Islands, Spain). Subjects were fully vaccinated at the time of sample collection. Subjects with underlying diseases or those receiving medical treatment were excluded from the study.

Upper-airway samples (Oropharyngeal/saliva) and paired-peripheral blood samples were collected from these subjects on the same day and during the first seven days of infection, which usually coincided with the beginning of symptoms. The samples were stored at −80 °C until their analysis for this study.

This study was conducted following the Declaration of Helsinki. The Hospital Universitario de Canarias ethical committee board (approval code: CHUC B1947, 2021, approval date 9 September 2021) approved this study, and written informed consent was obtained from all participants.

### 2.2. Gene Expression Study

Genes related to the immune and inflammatory response were analysed: *CCL5*, *IFI6*, *OAS1*, *IRF9*, *IL1B*, and *TGFB1*. These genes were selected based on relevant results in the literature and according to our previous findings (Gajate-Arenas et al. 2023).

The RNA isolation from upper airway samples was performed using Maxwell^R^ 16 Viral Total Nucleic Acid Purification Kit (Promega, Madison, WI, USA), and QIAamp^R^ RNA mini kit (Qiagen, Hilden, Germany). The RNA extraction from whole blood samples was performed using TRIzol™ Reagent (InvitrogenTM, Carlsbad, CA, USA). The RNA quality was determined by NanoDrop Lite (ThermoFisher Scientific, Waltham, MA, USA). A two-step RT-qPCR was set up for the relative gene expression analysis. cDNA synthesis was carried out using the High-Capacity cDNA Reverse Transcription Kit (Applied Biosystems, Waltham, MA USA), and following the manufacturer’s instructions. This was followed by a qPCR using the TaqMan™ Fast Advanced Master Mix and TaqMan™ Gene Expression Assays (ThermoFisher Scientific, Applied Biosystem, Waltham, MA, USA). The information about primers is included in Supplementary Table S1. The reaction was performed in a real-time thermocycler QuantStudio 5 (ThermoFisher Scientific, Applied Biosystem, Waltham, MA, USA). Each reaction was performed in duplicate, and the experiment was set up in 40 cycles. The *ACTB* gene was used for data normalisation. The relative expression analysis was determined using the comparative threshold method 2^ΔΔ^Ct [34].

### 2.3. Statistical Analysis

Continuous variables were described using means and standard deviation or median and percentiles (P_25_–P_75_) when not normally distributed.

The gene expression levels for blood and upper-airway samples were compared through paired comparisons. The differences in gene expression were found to be non-normally distributed for all genes examined. Consequently, a nonparametric test was conducted as appropriate. When the distribution of differences is symmetrically shaped, a Wilcoxon signed-rank test was provided. Alternatively, if the distribution was not symmetric, the sign test was applied. Spearman’s rank correlation coefficient was used to analyse the correlation between studied variables.

To compare the frequency of gene expression between the studied samples, McNemar’s test was used. A chi-square with continuity correction or Fisher’s exact test analysis, as appropriate, was performed to test differences in terms of sex.

SPSS v25.0 (IBM Statistics) was used for statistical analyses, and GraphPad Prism v9.0.0 (Dotmatics) was used for graphics generation. Two-tailed *p*-values < 0.05 were considered significant.

## 3. Results

A total of 25 individuals infected with SARS-CoV-2 with mild symptoms were analysed. The group consisted of 14 men and 11 women. The average age of men was 46.50 years and for women, this average was 40.09 years. Non-significant differences between groups were found (*p* = 0.211). The mean viral load Ct value was 28.76 ± 5.34 in the analysed cohort.

**Figure 1:**
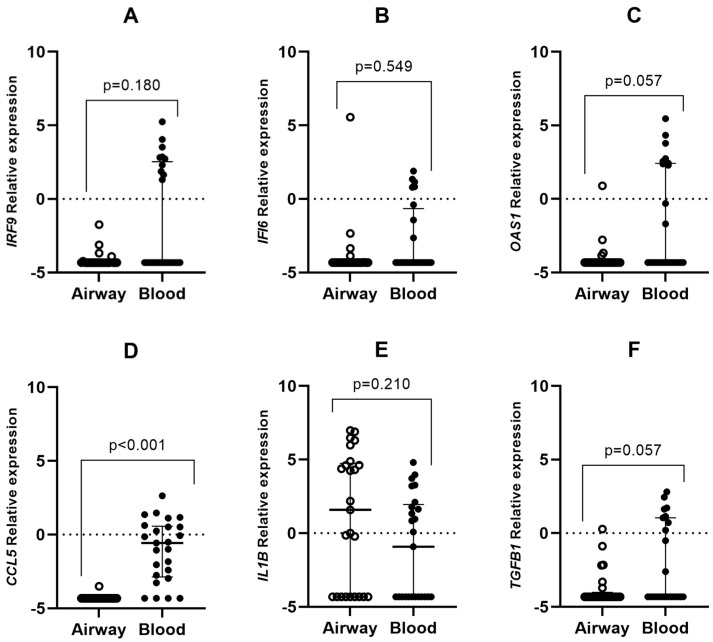
Differential gene expression between peripheral blood samples and upper-airway samples from mild COVID-19 individuals. Lines represent the median with an interquartile range. (**A**) *IRF9*, (**B**) *IFI6*, (**C**) *OAS1*, (**D**) *CCL5*, (**E**) *IL1B,* and (**F**) *TGFB1*. *p*-values < 0.05 were considered significant (Wilcoxon signed-rank test or sign test).

The expression of *CCL5*, *IFI6*, *OAS1*, *IRF9*, *IL1B*, and *TGFB1* genes was determined in both types of tissues in all the included participants. *CCL5* expression was significantly different between both sample types; blood samples expressed higher levels of this gene when compared to upper airway samples (Figure 1). Moreover, the expression ratio was higher in the blood than in the upper airways in *CCL5* (*p* < 0.001). The expression of the other genes was similar between the different types of tissue in the same individual. Furthermore, a correlation between viral load and *IFI6* expression in the upper airway was observed (r = 0.455; *p* = 0.033). Non-significant relationships were found between viral load and the levels of expression of other genes.

**Figure 2:**
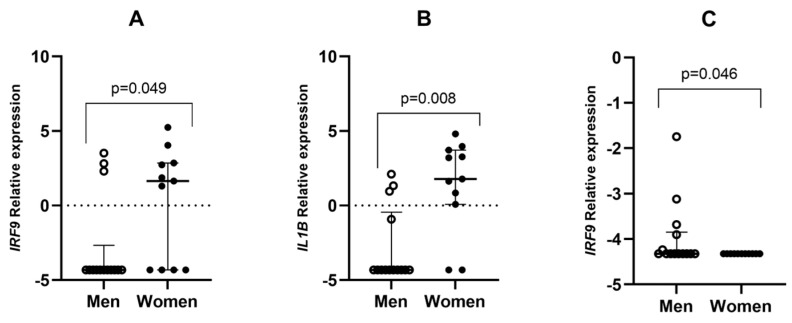
Differential gene expression between men and women in different tissues. (**A**,**B**) represent *IRF9* and *IL1B* expression, respectively, in blood samples. (**C**) represents *IRF9* expression in upper airway samples. Lines represent the median with an interquartile range. *p*-values < 0.05 were considered significant (McNemar’s test).

On the other hand, gene expression differences were found between sexes for *IL1B* and *IRF9* (Figure 2). These genes were proportionally higher expressed in the peripheral blood of women when compared to men (Figure 2). Nevertheless, men showed higher expression ratios of *IRF9* in the upper airway than women (*p* = 0.046).

## 4. Discussion

COVID-19 susceptibility depends on multiple factors, from clinical features to genetic factors [16,35,36]. SARS-CoV-2 infection starts in the upper airway, and it can spread to other tissues due to its tropism, promoting different responses in cells. In the present study, we analyse the differential expression of immune-related genes in upper-airway and peripheral blood samples in individuals with COVID-19 with mild symptoms of the disease.

Of all the genes analysed, only *CCL5* showed a differential expression between the blood and the upper airway from the same individuals. Specifically, higher levels of *CCL5* expression were found in blood samples of infected subjects. Our results are in line with those found by Martins et al. 2022 and Moratto et al. 2020, who reported high expression levels of *CCL5* in peripheral blood and plasma samples from COVID-19 patients compared to subjects without the infection [37,38]. However, the role of *CCL5* is not exempted from controversy. For example, it has been seen that higher levels of *CCL5* are associated with worse outcomes, amplifying the inflammation process, and promoting the development of acute respiratory distress syndrome (ARDS) [37,39,40]. Nevertheless, Perez-Garcia et al. 2022 reported that high expression levels of *CCL5* in the upper airway and a low viral load were associated with better outcomes, turning *CCL5* into a good predictor of COVID-19 severity [41]. Moreover, a gene expression study on peripheral blood samples showed that the increase in *CCL5* in the early stage of the infection might prevent severe COVID-19 [42].

The key to this controversy could be in the balance of the response. As we mentioned before, CCL5 induces the migration and recruitment of immune cells [26]. A strongly increased expression of chemokines, like CCL5, may cause the over-recruitment of immune cells and promote damage in the lung. For instance, Gang Xu et al. 2020 compared the expression of peripheral blood mononuclear cells and broncho-alveolar lavage fluid cells in COVID-19 patients and found that monocytes and macrophages from broncho-alveolar lavage fluid produce higher levels of chemokines, among them CCL5, than their blood counterparts, especially in severe cases [43]. Moreover, the over-expression of *CCL5* in the lower airway can be translated into low levels of *CCL5* in peripheral blood in severe cases, due to the migration of immune cells to the lungs [38]. Further studies are warranted to address this specific point. We want to highlight that CCL5′s role is essential for an effective immune response; defects in CCL5 expression lead to failure in infection control [44]. The influence this chemokine exerts in the immune system influx through different tissues makes it a suitable biomarker for COVID-19 prognosis and potentially a valuable ally for the personalised treatment of patients.

Viral load has been reported as crucial for detecting active infections and monitoring disease progression [45,46]. In our study, IFI-6 was found to be associated with the viral load in SARS-CoV-2-infected individuals. *IFI6* is a small protein that stabilises the mitochondrial function that leads to apoptosis discontinuation, and its expression has been related to viral inhibition. The study carried out by Meyer et al. 2015 shows that the *IFI6* expression inhibited the replication of hepatitis C virus in human hepatoma cells [47]. Moreover, Sajid M et al. 2021 demonstrated that *IFI6* expression can inhibit transcripts from the hepatitis B virus, decreasing viral replication [48]. Similarly, the overexpression of *IFI6*, together with the expression of other interferon-stimulated genes, reduced the replication of the Ebola virus in human embryonic kidney cells [49]. These facts show the capacity of this gene to reduce viral replication, which can explain why *IFI6* expression is related to viral load. In a previous study performed by our group, we observed that infected individuals with high viral loads presented an increased expression of *IFI6* in the upper airways [33]. Considering the current study and the above-mentioned findings, the relationship between *IFI6* expression and viral load should be analysed deeply in an extended cohort.

In the current study, similar expression levels of *IL1B* were found in the upper airways and peripheral blood of cases with mild COVID-19. This is consistent with the research of Lücke et al. 2023, who reported that high levels of IL1B in the blood were inversely associated with overall survival time [50]. However, the same study indicated opposite roles of intestinal in contrast to peripheral blood IL1B expression, suggesting the importance of tissue-specific analysis.

Concerning sex, it has been described that severity and mortality rates are higher in men than women. Sex-based differences could be influenced by many factors [11,51]. For example, X chromosome inactivation in females to maintain gene expression dosage in balance provides plasticity and adaptability in response to infections [52]. In the current study, a higher proportion of infected women showed increased expression of *IL1B* and *IRF9* in peripheral blood than infected men. The differences between sexes in the immune response are mainly due to genetic and hormonal factors that might alter the susceptibility and progression of the disease [53]. Women seem to be less susceptible to viral infections, which might be due to a higher activity of the innate immune response that promotes a faster virus recognition and consequently a higher synthesis of interferon type 1 and cytokines to control the infection [51]. This process could explain why we found an increased expression of *IL1B* and *IRF9* in women. It is important to clarify the possible role of IL1B in the context of SARS-CoV-2 infection. IL1B, a proinflammatory cytokine, has been found to increase in the bronchoalveolar lavage (BAL) fluid of COVID-19 patients compared to healthy controls. IL1B has been related to severe COVID-19 and has also been observed at higher levels in men [54,55]. Several studies suggest that persistently increased levels of IL-1β might contribute to the persistence of these symptoms [56]. On the other hand, low expression of *IL1B* has been reported in the early course of the infection [23]. Similarly, low levels of *IL1B* were observed in mild cases compared to controls in a previous study of our group [33]. The present study examines mild cases, where men showed low levels of *IL1B* in their blood in the early stages of the disease. Consequently, it is essential to consider the context of each patient for an accurate analysis of genes involved in the immune response, such as *IL1B* in different tissues. Additionally, a higher proportion of men showing increased levels of *IRF9* expression in the upper airway was observed in this study. Several studies reported families with genetic deficiencies of IRF9 and respiratory viral infections, demonstrating that this transcription factor is essential for the immune response controlling virus spread [57,58]. *IRF9* expression has been associated with better outcomes in viral infections, including COVID-19 [22,27,59]. We hypothesise that the expression of *IRF9* in the upper airway enhances pathogen control and promotes a coordinated local immune response, especially in men. Research in larger cohorts that delves into differences between sexes in the immune response is needed.

The present study has some limitations that are important to mention. First, our study focused on mild COVID-19 cases while severe cases might be necessary to expand our analysis and confirm some of the present findings. Secondly, our sample size is not very extensive; nevertheless, the availability of paired samples of different tissues of the same individual as in this study is usually an important challenge. Thirdly, studying patients with underlying conditions would have been valuable in assessing how these conditions influence gene expression concerning viral infection. An expanded cohort that categorises cases based on their comorbidities is essential for a comprehensive evaluation of the changes in immune and inflammatory response-related genes.

## 5. Conclusions

In conclusion, a higher expression of *CCL5* was found in the peripheral blood of mild COVID-19 cases in contrast to the upper airway. In COVID-19 cases, *CCL5* might play an important role in controlling the immune cell circulation at the first stage of infection, avoiding excessive recruitment in the airways. Furthermore, the expression of *IFI6* seems to be involved in controlling SARS-CoV-2 infection in the upper airway of mild cases. Sex differences are observed in the immune-related gene response in the early stages of the infection. The role of sex in this process is complex and requires in-depth study. Further studies in a larger cohort are necessary to corroborate the current findings and help advance personalised medicine.

## Data Availability

Data are available upon reasonable request. All data relevant to the study are included in the article or uploaded as additional information.

## Supplementary Materials

The following supporting information can be downloaded at: https://www.mdpi.com/article/10.3390/jpm14111099/s1, Table S1: Characteristic of the study genes.
