# Supplementary material for: Differential Inflammatory and Immune Response to Viral Infection in the Upper-Airway and Peripheral Blood of Mild COVID-19 Cases
Source: J Pers Med. 2024 Nov 9;14(11):1099. doi: 10.3390/jpm14111099 (PMC11595938; doi:10.3390/jpm14111099)
Supplement: Supplementary file 1 [file jpm-14-01099-s001.zip › jpm-3287492-supplementary.pdf]

Table S1. Primers and probes assays used for gene expression study.

Genes	Acronym	Reference Sequence *	Reference Assays **	Base Pairs
**C-C Motif Chemokine Ligand 5**	*CCL5*	NM_001278736	Hs00982282_m1	70
**Interferon Alpha Inducible Protein 6**	*IFI6*	NM_002038	Hs00242571_m1	115
**Transforming Growth Factor Beta 1**	*TGFB1*	NM_000660	Hs00998133_m1	63
**Interleukin 1 Beta**	*IL1B*	NM_000576	Hs01555410_m1	91
**Interferon Regulatory Factor 9**	*IRF9*	NM_006084	Hs00196051_m1	66
**2'-5'-Oligoadenylate Synthetase 1**	*OAS1*	NM_001032409	Hs00973635_m1	82
**Actin Beta**	*ACTB*	NM_001101	Hs01060665_g1	63

* Reference sequence from the GenBank database, NCBI (National Center from Biotechnology Information), NIH (National Library of Medicine), USA. ** Reference assays for gene expression analysis (Thermofisher Scientific, USA).
